# Circulating extracellular RNAs, myocardial remodeling, and heart failure in patients with acute coronary syndrome

**Published:** 2019-06-08

**Authors:** Khanh-Van Tran, Kahraman Tanriverdi, Gerard P. Aurigemma, Darleen Lessard, Mayank Sardana, Matthew Parker, Amir Shaikh, Matthew Gottbrecht, Zachary Milstone, Selim Tanriverdi, Olga Vitseva, John F. Keaney, Catarina I. Kiefe, David D. McManus, Jane E. Freedman

**Affiliations:** ^1^Department of Medicine, Health Sciences University of Massachusetts Medical School, Worcester, MA, USA; ^2^Population and Quantitative Health Sciences University of Massachusetts Medical School, Worcester, MA, USA

**Keywords:** Extracellular RNAs, Heart failure, Cardiac remodelling, Echocardiographic phenotypes, Biomarkers

## Abstract

**Background:**

Given high on-treatment mortality in heart failure (HF), identifying molecular pathways that underlie adverse cardiac remodeling may offer novel biomarkers and therapeutic avenues. Circulating extracellular RNAs (ex-RNAs) regulate important biological processes and are emerging as biomarkers of disease, but less is known about their role in the acute setting, particularly in the setting of HF.

**Methods:**

We examined the ex-RNA profiles of 296 acute coronary syndrome (ACS) survivors enrolled in the Transitions, Risks, and Actions in Coronary Events Center for Outcomes Research and Education Cohort. We measured 374 ex-RNAs selected *a priori*, based on previous findings from a large population study. We employed a two-step, mechanism-driven approach to identify ex-RNAs associated with echocardiographic phenotypes (left ventricular [LV] ejection fraction, LV mass, LV end-diastolic volume, left atrial [LA] dimension, and LA volume index) then tested relations of these ex-RNAs with prevalent HF (N=31, 10.5%). We performed further bioinformatics analysis of microRNA (miRNAs) predicted targets’ genes ontology categories and molecular pathways.

**Results:**

We identified 44 ex-RNAs associated with at least one echocardiographic phenotype associated with HF. Of these 44 exRNAs, miR-29-3p, miR-584-5p, and miR-1247-5p were also associated with prevalent HF. The three microRNAs were implicated in the regulation p53 and transforming growth factor-β signaling pathways and predicted to be involved in cardiac fibrosis and cell death; miRNA predicted targets were enriched in gene ontology categories including several involving the extracellular matrix and cellular differentiation.

**Conclusions:**

Among ACS survivors, we observed that miR-29-3p, miR-584-5p, and miR-1247-5p were associated with both echocardiographic markers of cardiac remodeling and prevalent HF.

**Relevance for Patients:**

miR-29c-3p, miR-584-5p, and miR-1247-5p were associated with echocardiographic phenotypes and prevalent HF and are potential biomarkers for adverse cardiac remodeling in HF.

## 1. Introduction

Heart failure (HF) is a rapidly rising public health problem that affects more than 37 million people worldwide with high morbidity and mortality [1,2]. It is a systemic disease, in which structural, neurohumoral, cellular, and molecular mechanisms that maintain physiological functions become pathological [3,4]. Together, these dysfunctional processes lead to increased cardiac remodeling, circulation redistribution, and volume overload [5]. Key to prevention and treatment of HF is the understanding of maladaptive cellular responses that lead to this disease. In particular, there is an urgent need to better understand the molecular mechanisms by which this pathological response is coordinated.

Small noncoding RNAs regulate signaling pathways that dictate physiological as well as pathological responses to stress. MicroRNAs (miRNAs) are small noncoding RNAs that modulate cardiac differentiation, proliferation, maturation, and pathological remodeling responses to environmental stimuli [6,7]. Extracellular RNAs (ex-RNAs) are endogenous small noncoding RNAs that exist in the plasma with remarkable stability and may reflect cellular states and cellular communication [8]. Although there are several reports implicating ex-RNAs in HF [9-11], the observations are biased due to the study of only a limited number of miRNAs. In a broader and unbiased screen of circulating ex-RNAs, specific miRNAs were found to be expressed in the setting of HF; however, the expression of ex-RNAs in acute clinical settings remains unknown [12]. Data illustrating the expression of plasma ex-RNAs in the acute clinical setting could provide relevant ex-RNA biomarkers and shed light on the molecular mechanisms underlying clinical HF.

Transthoracic echocardiography is a useful noninvasive technique to assess cardiac function and for prognostication of HF [13]. Cardiac remodeling as measured by enlarged cardiac chamber size, lower left ventricular ejection fraction (LVEF) or higher LV mass (LV mass) is associated with the incidence of HF [14-16]. Furthermore, changes in echocardiographic phenotypes are associated with rapid progression of the disease [17]. The high utility of echocardiographic parameters in the evaluation and prognostication of HF is due to its ability to define structural processes underpinning pathological cardiac remodeling. Although echocardiographic phenotypes associated with HF are well known, the molecular basis for pathological cardiac remodeling is less understood.

To better understand the signaling pathways activated in HF, we examined ex-RNAs relevant to cardiac remodeling as well as clinical HF in a hospitalized patient population. We employed a two-step analysis model that leveraged echocardiographic phenotypes associated with cardiac remodeling and prevalent HF in acute coronary syndrome (ACS) survivors from the Transitions, Risks, and Action in Coronary Events Center for Outcomes Research and Education (TRACE-CORE) cohort. In this study, we applied a mechanism-based framework to identify promising candidate ex-RNAs in the acute clinical setting to shed light on the molecular processes that drive HF.

## 2. Materials and Methods

### 2.1 Study population

**Figure 1:**
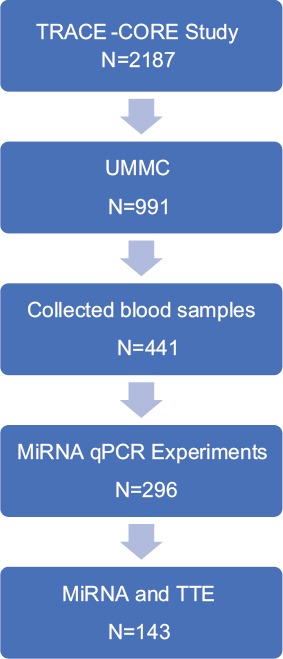
Sample selection for the analyses from the Transitions, Risks, and Action in Coronary Events Center for Outcomes Research and Education study.

Details of the design, participant recruitment, interview processes, and medical record abstraction procedures used in TRACE-CORE study have previously been reported [18,19]. In brief, TRACE-CORE used a 6-site prospective cohort design to follow 2187 patients discharged after an ACS hospitalization from April 2011 to May 2013 (Figure 1). Sites in Central Massachusetts included two academic teaching hospitals and a large community hospital. The other sites included two hospitals affiliated with a managed care organization in Atlanta, GA, and an academic medical center. At the sites in Central Massachusetts, 411 blood samples were collected, processed as described previously, and plasma was stored in −80°C [8,20]. Of the plasma collected, 296 were of sufficient quality for RNA extraction and qPCR experiment. The institutional review boards at each participating recruitment site approved this study. All participants provided written informed consent.

### 2.2 Ascertainment of HF

Trained study staff abstracted participants’ baseline demographic, clinical, laboratory, and electrocardiographic data and in-hospital clinical complications from available hospital medical records. Comorbidities present at the time of hospital admission were identified from each participant’s admission history and physical examination. Any patient with documentation of HF by a trained medical provider was considered as having prevalent HF.

### 2.3 ex-RNA selection and profiling

As part of a transcriptomic profiling study, we collected venous blood samples from 296 TRACE-CORE participants’ in-hospital admission. The methods for processing blood samples, storing plasma samples, and RNA isolation have previously been described [20]. We have previously published methods for quantification of ex-RNAs, which included miRNAs and small nucleolar RNAs (*snoRNAs*) [8]. ex-RNAs were selected *a priori*, based on previously generated data from the Framingham Heart Study [8]. The ex-RNA profiling of plasma was performed at the High-Throughput Gene Expression and Biomarker Core Laboratory at the University of Massachusetts Medical School. ex-RNA levels reported in quantification cycles (C_q_) where higher C_q_ values reflect lower ex-RNA levels. This approach yielded 331 miRNAs and 43 snoRNAs. Full details of ex-RNA profiling are described in supplementary information (Supplementary Table 1).

### 2.4 Echocardiographic measurements

Complete two-dimensional (2D) echocardiograms were performed during hospitalization. Ejection fraction, 2D volumes, and linear dimensions were measured according to ASE guidelines [21]. We quantified LV mass, LVEF, LV end diastolic (LVED) volume, left atrial (LA) volume, and LA volume index (LAVI) (Table 1). In brief, Simpson’s biplane summation of disks method was used to make measurements in apical 2-chamber and 4-chamber views. LV mass was calculated by LV mass=0.8 (1.04[LVID+PWTd+SWTd]^3^–[LVID]^3^)+0.6g [22].

**Table 1:** Characteristics of TRACE-CORE participants included in the analytic sample.

Characteristics	No heart failure (*n*=265)	Heart Failure (N=31)	P-value
Age, mean SD	63±11	68±13	<0.01

Female	34%	23%	0.19
Race (Caucasian)	96%	100%	0.32
Height (inches)	69±14	68±5	0.29
Weight (lbs)	187±46	191±57	0.66
Body mass index (kg/m^2^)	29±6	30±5	0.79

**Social history**			

**Education**			
High school	38%	58%	
Some college	28%	26%	<0.01
College	34%	16%	
Married	68%	52%	0.08

**Risk factors**			

Hyperlipidemia	67%	77%	0.25
Myocardial infarction	25%	74%	<0.001
Anginal pectoris/CHD	23%	67%	<0.001
Type 2 diabetes mellitus	28%	32%	0.65
Stroke/TIA	2%	3%	0.64
Atrial fibrillation	7%	29%	<0.001
Hypertension	68%	90%	<0.01

**Heart failure symptoms**			

Angina	71%	68%	0.74
Dyspnea	37%	52%	0.11

**Seattle angina questionnaire**			

Physical limitation	83.9±21.6	64.7±28.2	<0.01
Angina stability	43.1±27.4	44.6±31.9	0.81
Angina frequency	75.4±23.7	68.3±22.8	0.12
Treatment satisfaction	94±11.5	91.7±9.9	0.30
Quality of life	64.8±25.9	56.3±27.5	0.09

**Admission medications**			

Aspirin	45%	81%	<0.001
Beta-blocker	38%	87%	<0.001
ACEI or ARB	36%	71%	<0.001
Statin	56%	84%	<0.01
Plavix	12%	26%	0.06
Coumadin	4%	26%	<0.001

**Physical activity**			

No physical activity	59%	77%	
<150 min/week	16%	13%	0.08
>150 min/week	25%	10%	
**Acute coronary syndrome category**			

ST-elevation myocardial infarction	28%	10%	<0.05

**Physiological factors**			

Heart rate (beats per minute)	79±21	84±25	0.17
Systolic blood pressure (mmHg)	141±24	129±29	<0.01
Diastolic blood pressure (mmHg)	80±17	70±14	<0.01
Respiratory rate (breaths per minute)	18±4	19±3	0.51

**Electrocardiogram**			

QRS duration	95±18	120±34	<0.01
PR interval	164±30	182±25	<0.01

**Lab values**			

Troponin peak	25.8±36.7	6.0±17.1	<0.001
Total cholesterol	175.4±46.1	130.1±36.2	<0.01
Brain natriuretic peptide	581.8±846.3	758.7±665.9	0.55
Creatinine	1.1±0.4	1.8±1.0	<0.01
Hemoglobin	11.7±2.2	10.8±2.2	<0.05
Sodium	136±3	135±4	0.32

Echocardiographic phenotype*			

LV ejection fraction	53.7±13.0	45.0±8.8	0.07
LV mass	180.0±58.1	230.3±77.0	<0.05
LAVI=LAVavg/BSA	23.0±8.9	32.0±9.0	<0.01
LA volume	45.7±19.3	64.2±24.8	<0.01
LV end diastolic volume	83.5±38.3	132±51.4	<0.01

CHD: Coronary heart disease, TIA: Transient ischemic attack, ACEi: Angiotensin-converting enzyme inhibitors, ARB: Angiotensin II receptor blockers, LV: Left ventricle, LA: Left atrium, LAVI: Left atrial volume index, LAVavg/BSA: Average left atrial volume/body surface area.

* Echocardiographic phenotypes were characterized in a subset of patients (*n*=143) where TTE were available, TRACE-CORE: Transitions, Risks, and Actions in Coronary Events Center for Outcomes Research and Education

### 2.5 Statistical analysis

A two-step analysis model was used to leverage echocardiographic phenotypes to identify candidate ex-RNAs and then examining ex-RNAs identified and prevalent HF. In Step 1, we examined the relations between ex-RNAs with one or more echocardiographic phenotypes (Table 2, Supplementary Table 2). In Step 2, we examined the associations of ex-RNAs identified from Step 1 with prevalent HF (Table 3). Of note, the number of participants in each step differed as we did not have echocardiographic data available for all participants with plasma ex-RNA data. There are 143 cases with both ex-RNA and echocardiographic data in our TRACE-CORE cohort (Figure 1). We used this group to determine the ex-RNAs significantly related to one or more echo parameters. Using this significant list of ex-RNAs, we queried for a relationship with prevalent HF on the full 296 cases with ex-RNA data.

**Table 2:** ex-RNAs associated with echocardiographic phenotypes.

ex-RNA	No heart failure				Heart failure			
	
	*n*	Mean (1/Cq)	Median (1/Cq)	Std. Dev	N	Mean (1/Cq)	Median (1/Cq)	Standard deviation
hsa_miR_10a_5p	73	0.0526	0.0490	0.0216	10	0.0623	0.0484	0.0449

hsa_miR_10b_5p	111	0.0529	0.0519	0.0064	13	0.0531	0.0539	0.0036

hsa_miR_1246	263	0.0699	0.0689	0.0079	31	0.0695	0.0691	0.0053

hsa_miR_1247_5p	198	0.0533	0.0513	0.0146	25	0.0500	0.0496	0.0022

hsa_miR_1271_5p	8	0.0894	0.0522	0.0707	1	0.0458	0.0458	.

hsa_miR_142_5p	153	0.0548	0.0540	0.0129	15	0.0541	0.0517	0.0105

hsa_miR_144_5p	93	0.0538	0.0500	0.0340	9	0.0493	0.0495	0.0014

hsa_miR_148b_3p	192	0.0540	0.0537	0.0045	24	0.0528	0.0518	0.0039

hsa_miR_152_3p	118	0.0544	0.0528	0.0153	12	0.0546	0.0555	0.0038

hsa_miR_17_3p	39	0.0570	0.0484	0.0458	3	0.0484	0.0472	0.0028

hsa_miR_185_3p	12	0.0808	0.0470	0.0785	1	0.0457	0.0457	.

hsa_miR_186_5p	108	0.0495	0.0493	0.0027	14	0.0495	0.0494	0.0024

hsa_miR_190a_3p	30	0.0548	0.0482	0.0259	0	.	.	.

hsa_miR_200b_3p	40	0.0543	0.0476	0.0258	4	0.0716	0.0636	0.0297

hsa_miR_210_3p	65	0.0484	0.0481	0.0021	4	0.0471	0.0470	0.0011

hsa_miR_2110	63	0.0493	0.0483	0.0072	9	0.0487	0.0480	0.0024

hsa_miR_212_3p	18	0.0596	0.0464	0.0517	2	0.0465	0.0465	0.0011

hsa_miR_224_5p	90	0.0491	0.0484	0.0030	8	0.0492	0.0495	0.0026

hsa_miR_29b_3p	106	0.0511	0.0492	0.0148	5	0.0486	0.0487	0.0019

hsa_miR_29c_3p	157	0.0517	0.0512	0.0035	15	0.0497	0.0489	0.0035

hsa_miR_29c_5p	262	0.0589	0.0612	0.0057	31	0.0570	0.0545	0.0064

hsa_miR_337_3p	54	0.0540	0.0493	0.0212	1	0.0540	0.0540	.

hsa_miR_342_5p	25	0.0479	0.0474	0.0025	4	0.0860	0.0460	0.0802

hsa_miR_34a_3p	44	0.0481	0.0477	0.0038	5	0.0481	0.0473	0.0018

hsa_miR_424_3p	15	0.0618	0.0481	0.0539	1	0.0480	0.0480	.

hsa_miR_425_5p	67	0.0511	0.0494	0.0086	7	0.0541	0.0484	0.0117

hsa_miR_4446_3p	253	0.0577	0.0605	0.0067	29	0.0554	0.0523	0.0065

hsa_miR_450b_5p	36	0.0630	0.0482	0.0414	5	0.0501	0.0504	0.0042

hsa_miR_454_3p	51	0.0601	0.0487	0.0342	3	0.0494	0.0479	0.0031

hsa_miR_4770	34	0.0730	0.0495	0.0497	4	0.0475	0.0469	0.0023

hsa_miR_494_3p	26	0.0571	0.0476	0.0323	2	0.0598	0.0598	0.0146

hsa_miR_497_5p	62	0.0509	0.0491	0.0085	7	0.0494	0.0490	0.0031

hsa_miR_532_5p	39	0.0591	0.0477	0.0377	2	0.0801	0.0801	0.0440

hsa_miR_545_5p	12	0.0850	0.0497	0.0636	0	.	.	.

hsa_miR_548d_3p	32	0.0475	0.0472	0.0014	2	0.0481	0.0481	0.0009

hsa_miR_584_5p	159	0.0537	0.0510	0.0284	22	0.0492	0.0487	0.0024

hsa_miR_590_3p	20	0.0503	0.0486	0.0058	0	.	.	.

hsa_miR_596	13	0.0480	0.0480	0.0014	1	0.0474	0.0474	.

hsa_miR_642a_5p	16	0.1061	0.0972	0.0882	1	0.0479	0.0479	.

hsa_miR_656_3p	203	0.0587	0.0613	0.0078	22	0.0558	0.0547	0.0088

hsa_miR_6803_3p	41	0.0493	0.0483	0.0076	5	0.0990	0.0467	0.1154

hsa_miR_877_3p	71	0.0523	0.0504	0.0135	10	0.0562	0.0518	0.0159

hsa_miR_885_5p	67	0.0526	0.0486	0.0187	8	0.0641	0.0480	0.0350

hsa_miR_9_3p	78	0.0510	0.0498	0.0054	8	0.0558	0.0518	0.0137

**Table 3:** miRNAs significantly related to prevalent HF.

miRNA	*n*	Mean	Std	Estimate	Standard error	Prob Chi-square	Odds ratio	Lower CL	Upper CL	Raw*P* value	FDR*P* value
hsa_miR_1247_5p	223	19.3333	1.87668	0.4849	0.209	0.0203	1.624	1.078	2.446	0.0203	0.0485

hsa_miR_125b_5p	126	20.0642	1.00202	1.0689	0.4716	0.0234	2.912	1.156	7.34	0.0234	0.0485

hsa_miR_17_5p	207	19.2059	1.49941	0.3744	0.1849	0.0429	1.454	1.012	2.089	0.0429	0.0485

hsa_miR_181a_3p	216	19.0163	1.53413	0.587	0.2012	0.0035	1.799	1.212	2.668	0.0035	0.0185

hsa_miR_197_3p	237	19.8007	1.09519	0.454	0.2105	0.031	1.575	1.042	2.379	0.031	0.0485

hsa_miR_1_3p	92	19.8936	2.46961	1.3026	0.6502	0.0451	3.679	1.029	13.158	0.0451	0.0485

hsa_miR_200c_3p	31	20.0822	3.42561	-1.0268	0.4689	0.0285	0.358	0.143	0.898	0.0285	0.0485

hsa_miR_222_3p	221	18.9489	1.42712	0.3672	0.1803	0.0417	1.444	1.014	2.056	0.0417	0.0485

hsa_miR_26a_5p	261	17.5297	1.77419	0.3637	0.118	0.002	1.439	1.142	1.813	0.002	0.0185

hsa_miR_26b_5p	275	17.6456	1.65162	0.376	0.1272	0.0031	1.457	1.135	1.869	0.0031	0.0185

hsa_miR_27b_3p	226	19.1171	1.4456	0.351	0.1738	0.0434	1.421	1.01	1.997	0.0434	0.0485

hsa_miR_29c_3p	172	19.4854	1.28407	0.5425	0.2457	0.0272	1.72	1.063	2.784	0.0272	0.0485

hsa_miR_30a_5p	254	18.461	1.51398	0.3463	0.1443	0.0164	1.414	1.066	1.876	0.0164	0.0485

hsa_miR_30e_3p	143	19.6694	1.9183	-0.4287	0.2019	0.0337	0.651	0.439	0.967	0.0337	0.0485

hsa_miR_30e_5p	217	18.8058	1.45978	0.3254	0.1636	0.0467	1.385	1.005	1.908	0.0467	0.0485

hsa_miR_3613_3p	243	18.4669	1.6719	0.4435	0.1415	0.0017	1.558	1.181	2.056	0.0017	0.0185

hsa_miR_382_3p	96	20.389	1.12669	2.3048	1.168	0.0485	10.022	1.016	98.898	0.0485	0.0485

hsa_miR_495_3p	115	19.7469	1.49644	0.7446	0.3642	0.0409	2.106	1.031	4.299	0.0409	0.0485

hsa_miR_574_3p	108	20.0059	1.99826	1.103	0.5517	0.0456	3.013	1.022	8.884	0.0456	0.0485

hsa_miR_584_5p	181	19.6137	2.00572	0.4672	0.2098	0.026	1.595	1.058	2.407	0.026	0.0485

hsa_miR_7_5p	116	20.0465	1.01701	0.793	0.3373	0.0187	2.21	1.141	4.281	0.0187	0.048

Bolded are those significantly associated with echocardiographic phenotypes

For Step 1 of our analyses, we used ordinary least-squares linear regression to quantify associations between ex-RNA levels and one or more echocardiographic phenotypes in all participants. To account for multiple testing, we employed Bonferroni correction to establish a more restrictive threshold for defining statistical significance. We established a 5% false discovery rate (via the Benjamini–Hochberg false discovery rate approach) to screen associations between ex-RNAs and one or more echocardiographic phenotypes. The α for achieving significance was set at 0.05/340=0.000147 *a priori*. Note that, C_q_ represents a log measure of concentration, with exponentiation factor 2. In Step 2 of the analysis, we examined the associations of miRNAs identified from Step 1, with prevalent HF using a logistic regression model. Here, we used continuous C_q_ values to compare with prevalent HF (Table 3).

Differentially expressed miRNAs were analyzed using miRDB, an online database that captures miRNA and gene target interactions [23,24]. We acknowledge our use of the gene set enrichment analysis software, and molecular signature database (MSigDB) for gene ontology (GO) analysis [25]. The network and functional analyses were generated through the use Qiagen’s Ingenuity Pathway Analysis (IPA) [26]. All statistics were performed with SAS software version 9.3 (SAS Institute) with a 2-tailed *P*<0.05 as significant.

## 3. Results

### 3.1 Patient characteristics

The baseline demographic, clinical, and echocardiographic characteristics of the 296 study participants are outlined in Table 1. Study participants were middle-aged to older adults (mean age of 63±11 and 68±13 for the no HF [control group] vs. the HF group, respectively). There was a male predominance; women represented 34% and 23% of control and HF groups, respectively. The patients with HF had a significant higher history of myocardial infarction, coronary heart disease, hypertension, and atrial fibrillation (Table 1). The HF group was more likely to have experienced STEMI as compared with NSTEMI. Furthermore, QRS intervals tended to be longer in the group with HF. Patients with HF had lower LEVF and displayed a concordant trend of higher LV mass, LVED volume, LA volume, and LAVI. The mean LV mass in a patient with HF was 230±8.9 gm as compared to180±58.1 g those without prevalent HF (Table 1).

### 3.2 Association of ex-RNAs with echocardiographic phenotypes

A total of 374 ex-RNAs (331 miRNAs and 43 snoRNAs) were quantified in the plasma of TRACE-CORE participants included in our investigation. There were 44 ex-RNAs that associated with one or more echocardiographic parameters, independent of other clinical variables (Table 2). Three miRNAs that were associated with three or more echocardiographic traits, miR-190a-3p, miR-885-5p, and miR-596 (Supplementary Table 2).

### 3.3 Associations of ex-RNAs with prevalent HF

ex-RNAs associated with echocardiographic phenotypes (*n*=44 miRNAs) were investigated for their relationships with prevalent HF using logistic regression models. Three were significantly associated with prevalent HF, miR-29c-3p, miR-584-5p, and miR-1247-5p, all of which were inversely correlated. In general, lower ex-RNAs levels correlated with higher odds of having prevalent HF (Table 2). However, this is not consistent across all identified ex-RNAs. We found 21 ex-RNAs that associated with prevalent HF through unadjusted logistic regression modeling (Table 3).

### 3.4 Gene Targets of ex-RNAs associated with prevalent HF

**Figure 2:**
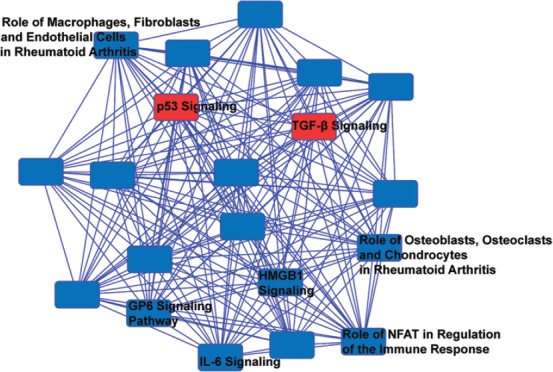
A network analysis of predicted targets of miR-29-3p, miR-584-5p, and miR-1247-5p as performed by ingenuity pathway analysis (IPA). Nodes represent signaling pathways, and lines are protein targets that are common between nodes. Nodes labeled with pathways are previously associated with inflammation, cardiac necrosis, and fibrosis. p53 and TGF-β signaling pathways are highlighted in red as they are pathways consistent with GO term analysis. Full list of top 20 predicted pathway by IPA is available in Supplementary Table 3.

**Figure 3:**
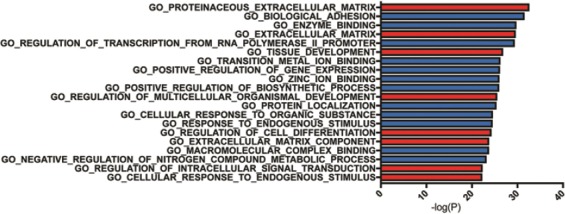
Gene ontology (GO) term analysis of predicted targets of miR-29-3p, miR-584-5p, and miR-1247-5p as performed by gene set enrichment analysis molecular signature database. Labeled in red is GO terms associated with p53 and transforming growth factor-β pathways and in blue are otherwise.

We investigated predicted targets of the three miRNAs associated with echocardiographic phenotypes and prevalent HF through miRDB. From this, 839 genes were predicted as targets for at least one miRNA. As miRNA are known to act in concert, we used the combined targets of miR-29c-3p, miR-584-5p, and miR-1247-5p to perform further analysis [6]. IPA was utilized to identify the molecular network and cellular toxicity pathways regulated by predicted targets. Overlapping canonical pathways were mapped to allow for visualization of the shared biological pathways through the common genes (Figure 2). The nodes identified included p53 signaling, transforming growth factor β (TGFβ) signaling, role of macrophages, fibroblasts and endothelial cells in rheumatoid arthritis, IL6 signaling, role of osteoblasts, osteoclasts and chondrocytes in rheumatoid arthritis, role of NFAT in regulation of the immune response, and mouse embryonic stem cell pluripotency (Figure 3, Supplementary Table 3). Highlighted in Figure 3 are pathways that were implicated in inflammation, fibrosis, and cell death; the complete list in the show in Supplementary Table 3. IPA identified predicted targets that are known to be involved in cellular toxicity based on previous reports. Table 4 lists the predicted targets as well as the cellular toxicity pathway, for example, cell death, cardiac fibrosis, p53, and TGFβ signaling. Notably, *DICER1, TGFB2, HDAC1, THBS4, THBS2*, and *PPARC1A* were among the targets identified. Gene ontology (GO) terms enrichment analysis using the MSigDB showed that miRNAs associated with echocardiographic phenotype and prevalent HF have strong associations with genes involved in the extracellular matrix, biological adhesion, and tissue development and cellular differentiation (Figure 2). We searched the literature for work exploring functions of miR-29c-3p, miR-584-5p, and miR-1247-5p (Supplementary Table 4). Dysregulation of miR-29c-3p has been implicated in cardiac development and cardiac fibrosis. miR-584-5p and miR-1247-5p have been implicated in the regulation of cellular proliferation and apoptosis in several malignancies (Supplementary Table 4).

**Table 4:** Cellular toxicity pathways implicated by predicted targets of miR 29c-3p, miR 584-5p, and miR 1247-5p.

Ingenuity toxicity pathway	-log (*P*-value)	Ratio	Gene
Cardiac necrosis/cell death	3.75	0.068	*FNG, THBS4, LEP, LIF, PPIF, UBE4B, TNFAIP3, MDM2, KRAS, DICER1, THBD, THBS2, PRKAA1, WISP1, NAMPT, GSK3B, CDK2, MCL1, CALCA, PPARGC1A*

p53 signaling	3.61	0.0982	*AKT2, TP53INP1, CCND2, TP63, GAB1, PIK3CG, PIK3R1, HDAC1, MDM2, GSK3B, CDK2*

Renal necrosis/cell death	3.23	0.0527	*PTHLH, EMP2, NF2, TNFAIP3, KRAS, PKN2, NFAT5, PRKAA1, NAMPT, PPM1A, AMER1, GNA13, GSK3B, CALB1, ZNF512B, MCL1, TRAF1, ITGB1, IFNG, TP53INP1, FOS, PEX5, GLIS2, CDC42, TMX1, CALCR, CDK2, CALCA, BIRC2*

TGF-b signaling	2.91	0.0938	*RAP2A, FOS, RUNX2, CDC42, BMPR1A, HDAC1, TGFB2, KRAS, TAB1*

TR/RXR activation	2.85	0.0918	*AKT2, GAB1, COL6A3, PIK3CG, PIK3R1, MDM2, G6PC, PPARGC1A, NCOA4*

Anti-apoptosis	2.79	0.156	*HDAC1, TMX1, TNFAIP3, MCL1, BIRC2*

Hepatic fibrosis	2.64	0.0857	*IFNG, LEP, COL6A3, COL4A3, THBS2, TGFB2, PDGFB, AHR, NID1*

Cell cycle: G1/S checkpoint regulation	2.57	0.101	*CCND2, HDAC1, CDK6, TGFB2, MDM2, GSK3B, CDK2*

Cardiac fibrosis	2.2	0.0605	*PTX3, ITGB1, IFNG, TRDN, TNFAIP3, CACNA1C, DICER1, NF1, BMPR1A, THBS2, GSK3B, DAG1, AHR*

VDR/RXR activation	1.71	0.0769	*IFNG, RUNX2, MXD1, TGFB2, CALB1, THBD*

Liver necrosis/cell death	1.63	0.0484	*IFNG, LIF, PIK3R1, DICER1, PDGFB, NPC1, FOS, NF1, PIK3CG, G6PC, GSK3B, AHR, PPARGC1A, BIRC2, MCL1*

Increases renal nephritis	1.63	0.0833	*IFNG, LEP, LIF, TRAF3IP2, COL4A3*

Liver proliferation	1.49	0.05	*ITGB1, IFNG, FOS, LEP, NFATC3, PIK3R1, HDAC1, PRKAA1, DICER1, GSK3B, CDK2, AHR*

Primary glomerulonephritis biomarker panel (human)	1.46	0.182	*SAMD4A, MCL1*

NF-kB signaling	1.39	0.0469	*RAP2A, IL36G, AKT2, TRAF3, GAB1, BMPR1A, PIK3CG, PIK3R1, HDAC1, TNFAIP3, KRAS, GSK3B, TAB1*

Mechanism of gene regulation by peroxisome proliferators via PPARa	1.34	0.0632	*FOS, PIK3R1, KRAS, PDGFB, TAB1, PPARGC1A*

Increases cardiac proliferation	1.33	0.08	*LEP, BMPR1A, WISP1, DICER1*

Increases renal proliferation	1.3	0.0541	*ITGB1, PTHLH, YBX3, WISP1, RNF144B, PTP4A1, PDGFB, CDK2*

Decreases depolarization of mitochondria and mitochondrial membrane	1.25	0.0938	*CSTB, MCL1, PPARGC1A*

TGF-b: Transforming growth factor-b

## 4. Discussion

In our investigation of ex-RNA profiles of 296 hospitalized ACS survivors in the TRACE-CORE Cohort, we identified 44 plasma ex-RNAs associated with one or more echocardiographic traits. Furthermore, three of these ex-RNAs, miR-29c-3p, miR-584-5p, and miR-1247-5p were associated with prevalent HF. While the association of miRNA and HF has been explored previously, our study uniquely examined the association between ex-RNA and HF in the acute clinical setting. We identified miR-29c-3p, miR-584-5p, and miR-1247-5p as regulators in cardiac remodeling and HF in patients hospitalized for ACS. Although miR-29 is a known to be downregulated in acute myocardial infarction and is a modulator of cardiac fibrosis [27], this is the 1^st^ time miR-584-5p and miR-1247-5p have been implicated as having a role in HF.

### 4.1 Echocardiographic phenotypes and cardiac remodeling in HF

Lower LVEF and concurrent higher in LV mass, LVED volume, LA volume, and LAVI reflect adverse cardiac remodeling [13,28,29]. Echocardiographic measures of cardiac remodeling have been shown to correlate with cellular hypertrophy as well as extracellular collagen deposition, metabolic dysregulation, and myocyte cell death [30]. Furthermore, changes in these characteristics prognosticate HF disease progression with unrivaled accuracy. Although HF involves several important pathological processes, we focused on cardiac remodeling as it is key in the evolution of HF. Here, we employed a mechanism-based approach to analyze the plasma miRome to tease out the complex components that contribute to cardiac remodeling in HF.

### 4.2 Association of ex-RNAs, cardiac remodeling, and HF

The association of ex-RNAs with structural remodeling has been explored recently [12]. However, few prior studies have examined quantitative echocardiographic phenotypes in humans in relation to plasma miRNA expression in the acute clinical setting. Consistent with previous data, our results revealed that miR-29c-3p is associated with cardiac remodeling [27]. We identified 44 ex-RNAs with statistically significant associations with the pre-specified echocardiographic HF endophenotypes, three of which were also associated with prevalent HF. Functional analysis of downstream targets supports existing evidence that HF is coordinated through several signaling pathways, most notably p53 and TFG-β signaling.

Cardiomyocyte cell death leads to cardiac dysfunction. Consistent with previous reports, we find that p53 signaling pathway is associated with prevalent HF [31]. p53 is a major inducer of apoptosis [32,33] which is upregulated in ventricular cardiomyocytes of patients with HF [31,34]. Promotion of p53 degradation prevents myocardial apoptosis [35]. We speculate that miR-29c-3p, miR-584-5p, and miR-1247-5p targets such as *CDK2* and *HDAC1* to regulate p53 signaling and that decrease of these regulators results in upregulation on p53, which leads to an increase in apoptosis [36-38].

One of the targets implicated in cardiac apoptosis is DICER1, a gene encoding a RNase III endonuclease essential for miRNA processing [39]. Chen et al. found that DICER is deceased in a patient with end-stage dilated cardiomyopathy HF requiring LV assist device (LVAD) compared to patients without HF [40]. Remarkably, DICER expression is increased post-LVAD transplantation, correlating with improved cardiac function. Furthermore, they found that cardiac-specific Dicer knockout in a mouse model leads to rapid progressive dilated cardiomyopathy, HF, and postnatal lethality [40]. Dicer mutant mice show aberrant expression of cardiac contractile proteins and profound sarcomere disarray. Existing literature supports our identification of *DICER1*, a predicted target of miRNA identified, as critical for cardiac structure and function.

Our analysis suggests that TGF-β plays an integral part in adult patients with HF. Cardiac cell death subsequently leads to tissue fibrosis, which is in part coordinated through the TGF-β signalizing pathway [41,42]. TGF-β2 is a predicted target of identified miRNAs along with other genes (Table 4). TGF-β has been shown to downregulate the miR-29 family, which, in turn, regulate expression of collagen Type I, alpha 1 and 2 and collagen Type III, alpha 1, all of which are involved in extracellular matrix production in the heart [27]. In addition, TGF-β1 has been shown to induce endothelial cells to undergo an endothelial-to-mesenchymal transition to contribute to cardiac fibrosis [43]. Serum TGF-β levels increase significantly in patients with hypertrophic cardiomyopathy [44]. Furthermore, myocardial TGF-β synthesis is consistently upregulated in animal models of HF [45,46].

GO categories analysis supported the hypothesis that miR-29c-3p, miR-584-5p, and miR-1247-5p have regulatory roles in cardiac remodeling through TGF-β. The top five GO categories are a proteinaceous extracellular matrix (GO:0005578), biological adhesion (GO:0022610), enzyme binding (GO:0031012), regulation of transcription from RNA polymerase promoter (GO:0006357), and tissue development (GO:0009888). Notably, there is a recurring theme of the GO term enrichment in extracellular matrix remodeling and cell differentiation, both of which has been shown to be regulated by TFG-β [47,48]. Together, our data support that miR-29c-3p, miR-584-5p, and miR-1247-5p affect cardiac remodeling structurally by influencing cell death and fibrosis, in part through the p53 and TFG-β signaling pathways.

Previously, we identified that miR-106b-5p, miR-17-5p, and miR-20a-5p 3 were associated with a reduction in long-term incident HF [12]. In our current analysis, we found that miR-17-5p was independently associated with prevalent HF. Wong et al. have examined the plasma miRome in patients with HF, HF with preserved ejection fraction (HFPEF), and HF with reduced ejection fraction (HFREF) and identified miRNAs associated with the clinical phenotypes [49]. We do not find an overlap between our ex-RNAs and those previously identified to be associated with HF. This could be due to the fact that the Singapore HF Outcomes and Phenotype (SHOP) cohort was a different racial and geographical cohort. Importantly, patients from the SHOP cohort were recruited from the ambulatory setting, whereas our TRACE-CORE cohort focused on patients in the hospitalized setting. Mick et al. examined ex-RNA associated with stroke or coronary heart disease [50]. There is no overlap in the ex-RNA identified to be associated with stroke, perhaps highlighting key differences between ACS and stroke.

### 4.3 Strength and limitations

Our study has several strengths. We examined ex-RNA associations with echocardiographic traits and HF in a well-characterized cohort study. TRACE-CORE is a cohort hospitalized ACS survivors, which uniquely provided the expression profiles of plasma ex-RNA in the acute clinical setting. In this study, our observations may reflect biomarker changes secondary to ACS rather than HF. However, we did not find any significant differences in ex-RNA due to AMI in our previous work [50]. As we used the same methodology to study ex-RNA in this study, the differential expression of ex-RNA observed is more likely secondary to HF status rather than ACS.

Our study has several shortcomings, among which is its relatively small sample size that is not racially or geographically diverse. We lack the power to examine whether these three miRNAs were associated with HF subtypes, HFPEF, or HFREF. Although we find that these miRs are associated with echophenotypes and HF, we have not located the sources or understand the mechanism by which they are transported in the blood. Further experiments at the bench are needed to explore these key questions to improve understanding of the molecular processes by which these miRs regulate HF.

## 5. Conclusions

In our analysis of echocardiographic, clinical, and ex-RNAs data from ACS survivors enrolled in the TRACE-CORE cohort, we observed that three ex-RNAs, miR-29c-3p, miR-584-5p, and miR-1247-5p were associated with echocardiographic phenotypes and prevalent HF. These ex-RNAs were predicted to mediate cardiac remodeling in part through the p53 and TFG-β signaling pathways. Further studies with a diverse cohort as well as basic experimentation are needed to validate our results. Our work establishes a mechanism-based framework for the identification of novel ex-RNAs biomarkers and downstream targets to attenuate cardiac remodeling that lead to HF.
